# Investigating differential effects of interpregnancy interval on pregnancy complications by country development status: protocol for systematic review and meta-analysis

**DOI:** 10.1186/s13643-026-03242-6

**Published:** 2026-07-29

**Authors:** Richard W. Wang, Aditya Ranganath, Hui Lin Lee, Yaduza Sivarasa, Ben D. A. Gorman, Caitlyn E. Flint, Carol A. Wang, Katherine A. Birchenall, Craig E. Pennell

**Affiliations:** 1 School of Medicine and Public Health, College of Health, Medicine and Wellbeing, The University of Newcastle, Newcastle, NSW Australia; 2 Reproduction and Family Health Research Program, Hunter Medical Research Institute, Newcastle, NSW Australia; 3 Mothers and Babies Research Centre, College of Health, Medicine and Wellbeing, The University of Newcastle, Newcastle, NSW Australia; 4 Maternity and Gynaecology, John Hunter Hospital, Newcastle, NSW Australia; 5 Department of Anaesthesia, Perioperative Medicine and Pain Management, Canberra Health Service, Canberra, ACT, Australia

**Keywords:** Interpregnancy interval, Birth interval, Preterm birth, Low birthweight, Small for gestational age, Fetal growth restriction, Perinatal mortality

## Abstract

**Background:**

Interpregnancy interval (IPI) has been reported to be associated with the risk of pregnancy complications. Short IPIs have been associated with higher risks of preterm birth, small for gestational age, low birthweight, fetal growth restriction, and perinatal mortality. However, there is limited information on how these associations differ according to the developmental status of a country. This review is therefore designed to examine the impact of the developmental status of a country on the relationship between IPI and perinatal complications.

**Methods:**

Studies will be identified through a comprehensive search of MEDLINE, EMBASE, Global Health, LILACS, and African Index Medicus databases, using key terms including “interpregnancy interval”, “birth interval”, “preterm birth”, “small for gestational age”, “fetal growth restriction”, and “perinatal mortality”. Studies to be included will be screened for data reporting on IPI and relevant outcomes. The study population will consist of women with two or more singleton births. The exposure of interest is short IPI, categorised as (1) < 24 monthsmonths; ≤ 18 months; and ≤ 12 months between the birth of a child and conception of the next pregnancy. These exposure groups will be compared against the World Health Organisation recommendation of an IPI of at least 24 months. Co-primary outcomes are preterm birth, small for gestational age, fetal growth restriction, low birthweight, and perinatal mortality. The developmental status of study populations will be categorised according to the World Bank income-based country classifications for low-, middle-, and high-income countries. Studies will be included if they have an observational design and excluded if they are systematic reviews with no new data. All studies will be screened, data extracted, and bias assessed by two independent authors, with all discrepancies being resolved by consensus or the advice of a third author. If studies have a low heterogeneity (*I*^2^ < 50%), the Mantel–Haenszel method, with a fixed-effects model, will be used to calculate pooled odds ratios (ORs) with 95% confidence intervals. If studies have high heterogeneity (*I*^2^ ≥ 50%) a random-effects (inverse-variance) model will be used instead.

**Discussion:**

Results from this review have the potential to guide future recommendations on optimal IPI in both high and low-resource countries. Further, they have the potential to assist in developing health strategies to target specific populations at risk from short IPI-related adverse pregnancy outcomes.

**Systematic review registration:**

PROSPERO CRD420251075289.

## Background

Interpregnancy interval (IPI) is defined as the period between the birth of one child and the conception of the next. A short IPI is associated with an increased risk of pregnancy complications [1]. To reduce this risk, the World Health Organisation (WHO) recommends that at least 24 months be allowed before attempting the next pregnancy to avoid adverse fetal outcomes [2]. Shorter IPIs (typically defined as less than 24 months) have been associated with increased risk of preterm birth (PTB), small for gestational age (SGA), low birth weight (LBW), fetal growth restriction (FGR), and perinatal mortality (PNM) [3].

Preterm birth (PTB) (birth before 37 completed weeks gestation) is one of the most significant challenges to perinatal health worldwide, and global estimates report 15 million PTB per year [4]. Despite best efforts to reduce preterm birth and develop prediction and management strategies [5–11], rates continue to rise globally [4–12]. PTB is the leading cause of death and disability in children under 5 years of age worldwide [11] and has substantial short-, medium-, and long-term sequelae related to physical and neurocognitive [13] development [14, 15]. Long-term complications of PTB include ischemic heart disease, heart failure, insulin resistance, chronic kidney disease, hypertension, obesity, and neurodevelopmental and social disability [16]. PTB across the gestational age spectrum is also associated with early mortality in adulthood; the earlier the PTB, the greater the early mortality [17]. There is an urgent need for interventions to reduce PTB in both high- and low-income settings. Similar to PTB, being SGA or having FGR is also associated with increased risk of adverse health outcomes across the life-course of the offspring. Short-term complications include increased perinatal morbidity and mortality [18]. Long term consequences include poor neurodevelopmental outcomes [19, 20] alongside an increased risk for non-communicable diseases in adulthood, including hypertension, insulin resistance, type 2 diabetes, coronary heart disease and stroke [21].

There has been debate in the literature regarding the mechanisms underlying the observed association between IPI and adverse pregnancy outcomes. It has been suggested that the impact of short IPI may be secondary to confounders such as maternal age, prior pregnancy outcome, or the socioeconomic status of a country [19, 20]. Short IPIs may compromise maternal readiness for subsequent pregnancies, increasing the risk of complications such as uterine rupture and uteroplacental bleeding disorders [21]. Suggested mechanisms for the risks associated with short IPI include cervical insufficiency, incomplete uterine remodeling postpartum, and maternal depletion syndrome [21]. The maternal depletion syndrome has been hypothesised to explain the observed reduced maternal and infant health in developing countries [26]. This hypothesis suggests that women with short IPIs do not have adequate time between pregnancies to replenish sufficient key micronutrients, such as iron and folate, deficiencies of which are associated with PTB and FGR [22]. Consequently, the adverse effects of short IPIs may be exacerbated by limited access to vitamin supplementation and widespread maternal malnutrition in low-income settings.

While prior reviews have reported clear associations between short IPIs and adverse pregnancy outcomes, the role of a country’s socioeconomic status in this relationship is unclear. Disparities in the prevalence of adverse fetal outcomes may be attributable to differences in socioeconomic status, healthcare infrastructure, and quality of primary care; factors more prevalent in lower income settings [23]. There exist systematic reviews and meta-analyses evaluating the short IPI effect on fetal outcomes in high-resource settings, although few have compared low-resource and high-resource countries [24]. This systematic review and meta-analysis will investigate how the relationship between IPI and pregnancy complications varies depending on a country’s economic status.

The specific aims of this study are to:

1. Assess whether there is a difference in the effect of short IPIs on the incidence of adverse pregnancy outcomes (including PTB, SGA, LBW, FGR, and PNM) between high-income and low- or middle- income settings; and,
2. To determine if there is an optimal IPI range that should be recommended within each setting to minimise the risk of adverse outcomes.

## Methods

### Study design

A systematic review and meta-analysis.

### Registration

The present protocol has been registered within the PROSPERO database (registration number CRD420251075289) and is being reported in accordance with the reporting guidance provided in the Preferred Reporting Items for Systematic Reviews and Meta-Analyses Protocols (PRISMA-P) [25] (Appendix 1).

### Eligibility criteria

The eligibility criteria for this systematic review will be pre-specified in the PROSPERO registration.

#### Participants

This review will consider all studies which include women with two or more singleton births. Studies that focus exclusively on women with previous adverse pregnancy outcomes or those who conceived through assisted reproductive technologies will be excluded. In studies that include women with 3 or more singleton births, only the second singleton birth will be included in the analysis where possible. Studies that have ambiguous birth ranks will be included in the meta-analysis and excluded for the sensitivity analysis.

#### Exposure

This review will include studies that assess the impact on pregnancy outcomes of short IPI. Short IPI will be defined as (1) less than 24 months, (2) less than or equal to 18 months, and (3) less than or equal to 12 months between the birth of a child and the conception of the next pregnancy. Only studies that report the interpregnancy interval as the time between the previous delivery and conception of the index pregnancy (inter-delivery interval minus gestational age of the index pregnancy) will be included in this review.

#### Comparator

This systematic review and meta-analysis will use an IPI of at least 24 months, the WHO definition [2], as the reference category.

To compare and assess the impact of short IPI in countries of different economic status, studies will be divided by the developmental status of the country of study participants as per the World.Bank’s country development classification at the time the study was conducted. Countries will be grouped into two categories based on gross national income (GNI) per capita: low- and middle-income countries versus high income countries [26]. For studies conducted over extended periods, country income classification will be based on the World Bank income classification at the midpoint year of data collection. Where a country has changed its income classification during the data collection window for an included study, the World Bank income classification representing the country at the midpoint of data collection will be used. For studies conducted prior to the advent of the World Bank Income classification in 1987, the income classification of the country in 1987 will be used. Subnational studies will be classified according to the national World Bank income classification of the country in which the study was conducted. Studies that cannot be classified or focus exclusively on a specific socioeconomic demographic will be excluded from the study, while studies that span classifications or were conducted prior to 1987 will be excluded in the sensitivity analysis. Where studies report results from multinational populations, data will be extracted and classified separately by county or excluded if this is not possible.

#### Outcomes

Co-primary outcomes are (1) PTB (defined as birth before 37 completed weeks of gestation), (2) SGA (birthweight less than the 10th percentile), (3) FGR, (4) LBW (birthweight less than 2500 g), and (5) PNM. Broadly, PNM encapsulates stillbirth and early neonatal mortality, though the precise definitions vary globally [27]. To avoid exclusion of data from LMICs all studies that included PNM as stillbirth or neonatal mortality will be included in the primary analysis. Secondary analyses will be conducted using the WHO definition (22 completed weeks gestation to 7 completed days after birth) [28] and with studies that did not define perinatal mortality. Finally, stillbirth and early neonatal mortality (0–7 days after birth) will be analysed as separate outcomes using the stillbirth definitions of greater than 20 weeks and greater than 28 weeks.

### Search strategy

Observational studies, such as cohort studies, cross-sectional reviews, and case–control studies, will be eligible for inclusion. Previous meta-analyses and systematic reviews that do not include original data will be excluded from the analysis.

The electronic databases MEDLINE, EMBASE, Global Health, LILACS, and African Index Medicus will be used to conduct the search. The search will include the following search terms: interpregnancy interval OR birth interval AND preterm birth OR small for gestational age OR fetal growth restriction OR perinatal mortality. There will be no restrictions placed on the length of study follow-up time, country or year; however, we will only be looking at studies published in English, and the search will be limited to studies focused on humans. Medical Subject Headings (MeSH) will be used. The detailed database search strategy is summarised in Appendix 2. The inclusion and exclusion criteria are summarised in Table 1.

**Table 1:** Inclusion and exclusion criteria

Domain	Inclusion criteria	Exclusion criteria
Population	Women with two or more singleton births	Studies that focus exclusively on women with previous adverse pregnancy outcomes or those who conceived through assisted reproductive technologies
Intervention/exposure	IPI less than 24 months IPI equal or less than 18 months IPI equal or less than 12 months	Studies that do not specify IPI or use an alternative IPI definition
Comparator	IPI of at least 24 months	
Outcomes	PTB SGA FGR LBW Perinatal mortality	Studies reporting alternative pregnancy outcomes
Study design	Cohort studies, cross-sectional reviews, and case–control studies	Non-human studies Systematic reviews unless they present new data
Language	Studies published in English	Non-English studies unless translation is available
Publication date	Nil restrictions	N/A
Setting	Studies conducted in countries defined clearly by World Bank income classification	N/A

### Study selection and data collection

Titles and abstracts identified through all sources will be exported into Rayyan software [29] and duplicates removed. Two authors will independently screen article titles and abstracts against the pre-specified inclusion/exclusion criteria. In the case of disagreement, authors will review the papers together; in the case of remaining disagreement, the article will be discussed with a third reviewer to resolve the situation. Included studies will then be reviewed in full and data extraction performed by two independent authors, and any discrepancies will be reviewed by a third and fourth author if required. All data will be extracted using a standardised electronic data extraction sheet. For cases with prior adverse events or inconsistent outcome reporting, two independent reviewers will assess for inclusion and where data cannot be formally analysed will be described. Once extracted, after reviewer agreement, data will be transferred into Review Manager version 5.3 data-analysis software [30].

The following data will be extracted:

- Study characteristics: authors; publication date; study design; country of study; sample size; confounding factors of participants; study size; source of data; data collection timeframe; funding and risk of bias.
- Exposure characteristics: IPI and number of participants in each exposure group.
- Outcomes: incidence, unadjusted or adjusted odds ratio of PTB, SGA, FGR, LBW, and perinatal mortality along with the precise definitions used for these outcomes.

Study characteristics will be examined to identify potential overlap between study populations. Where multiple publications use data from the same cohort with overlapping data-collection windows, the study with the most comprehensive dataset for each outcome will be prioritised for data extraction. Where different outcomes are reported from the same cohort, relevant data will be extracted without double counting.

In cases of heterogeneity in IPI categorisation across studies, reported intervals bands (e.g. < 6, 6–11, 12–17, 18–23, 24–59) will be pooled into the prespecified exposure (≤ 12, ≤ 18, and < 24) and comparator (≥ 24) groups where possible. Studies that are unable to be harmonised to our predefined groups will be excluded from the review. Sensitivity analyses will be conducted with the exclusion of studies with broad or non-standard interval groupings to assess the impact of pooling heterogeneous exposure intervals into the prespecified exposure groups.

The analytical hierarchy of this study will prioritise the extraction of the frequency of the outcomes in relation to our prespecified exposures, followed by unadjusted odds ratios. Where these are unavailable, adjusted odds ratios will be extracted provided they have included at least maternal age as a confounder. Unadjusted estimates are prioritised to maximise comparability across studies, given that adjusted estimates vary in selection of confounders used. However, given the importance of confounding in this literature, adjusted estimates will also be analysed in a separate subgroup analysis to assess consistency of findings. A subgroup analysis will be conducted to examine adjusted and unadjusted odds ratios independently.

### Data analysis

A meta-analysis will be performed by pooling studies using Review Manager software [30]. Results will be reported using odds ratios and 95% confidence intervals (CI), with IPI greater than or equal to 24 months as the reference group. Study heterogeneity will be assessed visually using forest plots. Further consideration of statistical heterogeneity will be quantified by calculating the *I*^*2*^ statistic. The *I*^*2*^ statistic quantifies variance among study results due to differences between study design rather than chance. If *I*^*2*^ is less than 50%, it indicates that studies are comparable with negligible to moderate heterogeneity [31] where a fixed-effects (Mantel–Haenszel) model is appropriate. An *I*^2^ of greater than or equal to 50% indicates potential substantial heterogeneity and a random-effects model (inverse-variance) will be used for subsequent analysis to account for variance within and between studies For all analyses with an *I*^2^ less than 50% a fixed-effects (Mantel–Haenszel) model will be used. A fixed-effects model will also be used for all outcomes with less than five studies [31]. Additionally, a subgroup analysis will be conducted using the same methodology to examine unadjusted and adjusted odds ratios independently. Sensitivity analyses will be conducted for each perinatal outcome where there are sufficient studies by removing low and average quality studies to examine their effect on the estimates.

For outcomes where data is available from ten or more publications, funnel plots and Egger’s regression test (for funnel plot asymmetry) will be performed to identify publication bias. Funnel plots will be plotted with effect sizes on the *x*-axis and standard errors on the y-axis to allow the visual assessment of funnel plot asymmetry. High-income and low-to-middle-income countries will be assessed separately.

Two independent authors will assess the risk of bias using the Newcastle–Ottawa Score (NOS) for cohort and case–control studies [32] and a modified version of the cohort NOS for cross-sectional studies (Appendix 3). This modification ensures that the cross-sectional NOS is applicable to the current study (i.e. scoring for the inclusion of the confounding factor of maternal age). The NOS seeks to address bias across three domains: exposure and comparator group selection, group comparability, and assessment of the outcome variable. Any variation in judgment between the authors of this study will be discussed and resolved. The NOS is scored out of nine points; studies scoring ≥ 8 will be considered high quality. The NOS criteria are specific to a study’s methodology, and the appropriate NOS version will be utilised depending on the study design, being cross-sectional, cohort, or case–control. No studies will be excluded from the primary analyses based on these scores. Sensitivity analyses will be performed to assess the robustness of the results after excluding studies with a score of seven or less.

Consistency and confidence of cumulative evidence in the primary outcome will be assessed independently by two authors using the Grading of Recommendations, Assessment, Development and Evaluations (GRADE) approach [33]. Evidence will be assessed for risk of bias, consistency, directness, precision, and publication bias. Overall grade will be weighted based on study sample size.

## Discussion

This systematic review and meta-analysis aim to evaluate the impact of IPI on perinatal outcomes and how these effects may vary according to the developmental status of a country or setting. Specifically, we will generate ORs and 95% CI for four pregnancy outcomes by IPI with countries grouped by economic status. This data will allow us to determine the minimum IPI that does not increase the odds of each adverse pregnancy outcome for high-income and low- and middle-income countries which could be used to revise clinical guidelines for best practice.

Findings from this review may also inform national and international guidelines relating to optimal birth spacing. Evidence on how the association between short IPI and adverse outcomes varies by country income status may also help inform family planning and contraceptive access programs, and antenatal counselling policies, especially in low- and middle-income countries.

The study has several strengths: first, the meta-analyses will include multiple publications for each obstetric outcome, many of which have large sample sizes. This is likely to result in relatively narrow confidence intervals for each outcome allowing the direct comparison of IPI durations within country developmental status groups. Further, the large number of studies included are likely to be populations from many countries increasing the generalisability of the results. However, there are limitations to our research paradigm, as it does not allow the examination of confounders or mechanisms as individual patient data is not available for the studies in this meta-analysis. Therefore, we will not be able to assess the biological and social mechanisms that mediate the increased risk of PTB, LBW and SGA in women with short IPI. Additionally, the inclusion of subnational studies may not reflect the national socioeconomic status. However, this is necessary due to the likely paucity of national datasets that link birth records with clinical information such as gestational age and perinatal outcomes. This limitation is mitigated by the exclusion of studies that focus on a specific socioeconomic demographic. Finally, the prioritisation of frequency data and unadjusted odds ratios over adjusted odds ratios maximises comparability between studies but limits the impact of confounding factors. This limitation is mitigated by the subgroup analysis that will examine adjusted and unadjusted odds ratios independently.

## Conclusion

This systematic review and meta-analysis aim to evaluate the impact of IPI on pregnancy outcomes and how these effects vary according to the developmental status of a country. Determining the optimal IPI for countries based on economic status will provide information to support the development of public health strategies to reduce the rate of PTB, SGA, FGR, LBW and perinatal mortality. By synthesising evidence from multiple studies, this review seeks to inform health authorities in creating country-specific guidelines on optimal IPI. Ultimately, this may help to reduce adverse outcomes associated with suboptimal IPI. We anticipate that the findings will benefit not only policymakers and healthcare providers but also women, families, and the broader community, providing additional information to support education and family planning for women in countries of different economic status.

## Data Availability

No datasets have been generated or analysed yet. Upon completion of the review, all data extracted will be made available on reasonable request.

## Appendix 1

### PRISMA-P checklist

**Table:**

Section and topic	Item no.	Checklist item	Line numbers of information reported
**Administrative information**			
Title:			
Identification	1a	Identify the report as a protocol of a systematic review	1–2
Update	1b	If the protocol is for an update of a previous systematic review, identify as such	N/A
Registration	2	If registered, provide the name of the registry (such as PROSPERO) and registration number	57
Authors:			
Contact	3a	Provide name, institutional affiliation, e-mail address of all protocol authors; provide physical mailing address of corresponding author	5–22
Contributions	3b	Describe contributions of protocol authors and identify the guarantor of the review	334–336
Amendments	4	If the protocol represents an amendment of a previously completed or published protocol, identify as such and list changes; otherwise, state plan for documenting important protocol amendments	N/A
Support:			
Sources	5a	Indicate sources of financial or other support for the review	N/A
Sponsor	5b	Provide name for the review funder and/or sponsor	N/A
Role of sponsor or funder	5c	Describe roles of funder(s), sponsor(s), and/or institution(s), if any, in developing the protocol	N/A
**Introduction**			
Rationale	6	Describe the rationale for the review in the context of what is already known	63–108
Objectives	7	Provide an explicit statement of the question(s) the review will address with reference to participants, interventions, comparators, and outcomes (PICO)	109–114
**Methods**			
Eligibility criteria	8	Specify the study characteristics (such as PICO, study design, setting, time frame) and report characteristics (such as years considered, language, publication status) to be used as criteria for eligibility for the review	124–183, Table 1 (185)
Information sources	9	Describe all intended information sources (such as electronic databases, contact with study authors, trial registers or other grey literature sources) with planned dates of coverage	176–183
Search strategy	10	Present draft of search strategy to be used for at least one electronic database, including planned limits, such that it could be repeated	438–498 (Appendix 2)
Study records:			
Data management	11a	Describe the mechanism(s) that will be used to manage records and data throughout the review	187–198
Selection process	11b	State the process that will be used for selecting studies (such as two independent reviewers) through each phase of the review (that is, screening, eligibility and inclusion in meta-analysis)	187–194
Data collection process	11c	Describe planned method of extracting data from reports (such as piloting forms, done independently, in duplicate), any processes for obtaining and confirming data from investigators	187–198
Data items	12	List and define all variables for which data will be sought (such as PICO items, funding sources), any pre-planned data assumptions and simplifications	199–224
Outcomes and prioritisation	13	List and define all outcomes for which data will be sought, including prioritisation of main and additional outcomes, with rationale	161–171
Risk of bias in individual studies	14	Describe anticipated methods for assessing risk of bias of individual studies, including whether this will be done at the outcome or study level, or both; state how this information will be used in data synthesis	247–259
Data synthesis	15a	Describe criteria under which study data will be quantitatively synthesised	225–241
	15b	If data are appropriate for quantitative synthesis, describe planned summary measures, methods of handling data and methods of combining data from studies, including any planned exploration of consistency (such as *I*^2^, Kendall’s τ)	225–241
	15c	Describe any proposed additional analyses (such as sensitivity or subgroup analyses, meta-regression)	167–171, 237–241
	15d	If quantitative synthesis is not appropriate, describe the type of summary planned	N/A
Meta-bias(es)	16	Specify any planned assessment of meta-bias(es) (such as publication bias across studies, selective reporting within studies)	242–246
Confidence in cumulative evidence	17	Describe how the strength of the body of evidence will be assessed (such as GRADE)	260–264

## Appendix 2

### Search term strategy

Search Strategy:

Ovid MEDLINE(R) ALL (1946 to September 12, 2025)

1. exp Birth Intervals/
2. pregnancy complications/or fetal growth retardation/or exp fetal membranes, premature rupture/or exp premature birth/or exp perinatal death/
3. Infant, Small for Gestational Age/
4. Infant, Low Birthweight/
5. (interpregnancy interval* or interpregnancy interval* or birth interval* or birth spacing* or pregnancy spacing* or pregnancy interval* or interconception interval* or inter-conception interval* or conception interval* or interdelivery interval* or interdelivery interval*).mp.
6. (preterm birth* or premature birth* or preterm deliver* or premature deliver* or early birth* or premature rupture*).mp.
7. ("small for gestational age" or SGA or "growth restricted infant*" or "fetal growth restriction" or "foetal growth restriction" or "fetal growth retardation" or "foetal growth restriction" or FGR or intrauterine-growth restriction or IUGR or low birthweight or LBW or underweight infant*).mp.
8. (perinatal death* or perinatal mortalit* or stillbirth* or neonatal death* or early-neonatal mortalit*).mp.
9. 1 or 5
10. 2 or 3 or 4 or 6 or 7 or 8
11. 9 and 10

Embase (1947 to 2025)

1. exp birth interval/or pregnancy spacing/or birth spacing/
2. (“interpregnancy interval” or "inter-pregnancy interval" or "birth interval" or "birth spacing" or "pregnancy spacing" or "pregnancy interval" or "interconception interval" or "inter-conception interval" or "conception interval" or "interdelivery interval" or "inter-delivery interval").mp.
3. exp "premature rupture of membranes"/or exp prematurity/or exp premature labor/or exp extremely premature birth/or exp very premature birth/
4. exp small for gestational age/or exp intrauterine growth retardation/or exp low birthweight/
5. exp perinatal death/or exp newborn death/
6. exp perinatal mortality/or exp newborn mortality/
7. (preterm birth* or premature birth* or preterm deliver* or premature deliver* or early birth* or premature rupture*).mp.
8. ("small for gestational age" or SGA or "growth restricted infant*" or "fetal growth restriction" or "foetal growth restriction" or "fetal growth retardation" or "foetal growth restriction" or FGR or intrauterine growth restriction or IUGR or low birthweight or LBW or underweight infant*).mp.
9. (perinatal death* or perinatal mortality* or stillbirth* or neonatal death* or early neonatal mortality*).mp.
10. 1 or 2
11. 3 or 4 or 5 or 6 or 7 or 8 or 9
12. 10 and 11
13. limit 12 to "remove medline records"

Global Health, LILACS and African Index Medicus

(“interpregnancy interval” OR “inter-pregnancy interval” OR “birth interval” OR “birth spacing” OR “pregnancy spacing” OR “pregnancy interval” OR “interconception interval” OR “inter-conception interval” OR “conception interval” OR “interdelivery interval” OR “inter-delivery interval” OR “pregnancy spacing” OR “birth spacing”) AND (“preterm birth” OR “premature birth” OR “preterm delivery” OR “premature delivery” OR “early birth” OR “premature rupture” OR “small for gestational age” OR “sga” OR “growth restricted infant” OR “fetal growth restriction” OR “foetal growth restriction” OR “fetal growth retardation” OR “foetal growth restriction” OR “fgr” OR “intrauterine growth restriction” OR “iugr” OR “low birth weight” OR “lbw” OR “underweight infant” OR “perinatal death” OR “perinatal mortality” OR “stillbirth” OR “neonatal death” OR “neonatal mortality”)

## Appendix 3

### Modified Newcastle-Ottawa Scale for quality assessment of cross-sectional studies

Selection: (Maximum 4 stars)

1. Representativeness of the sample:
  - Truly representative of the average in the target population. * (all subjects or random sampling)
  - Somewhat representative of the average in the target population. * (non-random sampling)
  - Selected group of users.
  - No description of the sampling strategy.
2. Sample size:
  - Justified and satisfactory.*
  - Not justified.
3. Non-respondents:
  - Comparability between respondents and non-respondents characteristics is established, and the response rate is satisfactory.*
  - The response rate is unsatisfactory, or the comparability between respondents and non-respondents is unsatisfactory.
  - No description of the response rate or the characteristics of the responders and the non-responders.
4. Ascertainment of the exposure (risk factor):
  - IPI defined as birth to conception (or calculated as birth-to-birth minus gestational age) and data related to IPI obtained from hospital records (or equivalent vital records).*
  - No description of the measurement tool.

Comparability: (Maximum 2 stars)

1. The subjects in different outcome groups are comparable, based on the study design or analysis. Confounding factors are controlled.
  - The study controls for the most important factor (maternal age).*
  - The study control for any additional factor. *

Outcome: (Maximum 3 stars)

1. Assessment of the outcome:
  - Independent blind assessment.**
  - Record linkage.**
  - Self report.*
  - No description.
2. Statistical test:
  - The statistical test used to analysze the data is clearly described and appropriate, and the measurement of the association is presented, including confidence intervals and the probability level (*p *value). *
  - The statistical test is not appropriate, not described or incomplete.

This is a modified version of the Newcastle-Ottawa Quality Assessment Scale for cohort studies that is bespoke for assessing the quality of cross-sectional studies of pregnancy outcomes in relation to interpregnancy interval.

## Abbreviations

CI: Confidence interval
EMBASE: Excerpta medica database
FGR: Fetal Growth Restriction
GRADE: Grading of Recommendations Assessment, Development, and Evaluation
IPI: Interpregnancy interval
LBW: Low birth weight
PRISMA-P: Preferred Reporting Items for Systematic Reviews and Meta-Analyses Protocol
PROSPERO: Prospective Register of Systematic Reviews
PTB: Preterm birth
NOS: Newcastle Ottawa Scale
SGA: Small for gestational age
WHO: World Health Organisation
